# Breakfast Intake and Factors Associated with Adherence to the Mediterranean Diet among Lebanese High School Adolescents

**DOI:** 10.1155/2019/2714286

**Published:** 2019-06-03

**Authors:** Rana Mounayar, Rana Jreij, Jennifer Hachem, Frida Abboud, Maya Tueni

**Affiliations:** ^1^Department of Chemistry and Biochemistry, Faculty of Sciences II, Lebanese University, P.O. Box 90656, Fanar, Lebanon; ^2^Department of Biology, Nutrition and Dietetics, Faculty of Sciences II, Lebanese University, P.O. Box 90656, Fanar, Lebanon; ^3^Department of Nutrition and Dietetics, Faculty of Public Health II, Lebanese University, P.O. Box 90656, Fanar, Lebanon

## Abstract

The Mediterranean diet has shown to have positive health impacts on metabolic diseases and cognitive performance. However, Mediterranean countries have witnessed a decreased adherence during the past years and the adoption of a more westernized dietary pattern. The aim of this study was to evaluate the association of Mediterranean diet adherence with sociodemographic, lifestyle, and anthropometric factors among Lebanese high school adolescents. Specifically, we aimed to analyse in this group the association between low adherence and breakfast intake. A cross-sectional survey was carried out on randomly selected students (268 boys and 332 girls), aged between 15 and 18 years old, from private and public schools in Beirut and Mount Lebanon. Data were obtained from self-administered questionnaires and anthropometric measurements. The Mediterranean Diet Quality Index for children and adolescents was used to assess the adherence to the Mediterranean diet. The results showed a high percentage of adolescents having a low adherence (43%), with girls (64.2%) having a significant (*p* < 0.001) higher adherence than boys (35.8%). Furthermore, the study proved that a lower adherence to the Mediterranean diet was significantly associated with a higher risk of obesity (15.5%), breakfast skipping (69.4%), and an unhealthy breakfast options (17.4%). Younger adolescents (47.4%), students from public schools (92.6%), and students with the highest grades (25.3%) had a significantly higher adherence to the Mediterranean diet than those from private schools (7.4%) and older adolescents (18.9%). In conclusion, results should plead for an increased awareness in Lebanese schools, supporting students to be more adherent to the Mediterranean diet, in order to prevent a further increase in metabolic diseases later in adulthood.

## 1. Introduction

The Mediterranean diet (MD) has largely been examined by several studies due to its positive health impacts [1, 2]. The traditional MD is a near-vegetarian diet with a high content in monounsaturated fatty acids mainly from olive oil, which constitutes its primary source of lipids, and thus it would be normal to expect the health benefits it provides [1, 3]. In fact, Mediterranean dietary patterns differ upon countries but are all characterized by an elevated intake of fruits and vegetables, breads and cereals (primarily whole grain), pulses and nuts, poultry and fish, along with an occasional intake of lean cuts of red meat (less than two servings per week), and a moderate alcohol intake (particularly wine) [3, 4].

The studies concerning the MD have often revealed that a greater adherence has been associated with a lower risk of obesity [5], cardiovascular diseases [6], and diabetes mellitus [1, 7], this association being partly due to the impact of the MD on abdominal adiposity [8] and on inflammatory markers [7]. Other than its known effect on body weight and metabolic diseases, studies have recently shown the beneficial impact of the MD on cognitive performance [4, 9]. Few studies have explained the reason behind this impact, but when measuring the effect of the MD on the brain function of adults and elderly, results have revealed that this therapeutic effect is primarily due to the B vitamin content and the antioxidants properties of the MD [4, 9].

Lebanon, among other Mediterranean countries, has been facing a nutritional transition during the past years [2, 10]. The traditional MD has progressively been fading due to the westernisation of food products and technology-driven culture [10, 11], encouraging for a new dietary pattern high in fat, refined sugar, and processed foods and causing a higher prevalence of metabolic diseases [12].

Since the risk of chronic diseases during adulthood is largely dependent on the dietary patterns adopted in adolescence [13], youth behaviors have become an area of increased attention over the past decade [14]. Studies have revealed that adolescents in several Mediterranean countries are facing a lower adherence to the Mediterranean diet (AMD), for example, the adolescents of Turkey [13], Crete [15], Italy [16], and Greece [17], while youth in Spain has been able to maintain a traditional MD [18, 19]. In Lebanon, adolescents have shown a low AMD and the adoption of a new western-like dietary pattern [11], which may constitute one of the main determinants of the increasing rates of obesity that Lebanese adolescents have been witnessing in the past years [20].

Many tools have been used to measure the AMD, and one of them is KIDMED test (Mediterranean Diet Quality Index for children and adolescents) that was developed and validated by Serra-Majem et al. [21], a tool specifically established to measure the adherence of children and adolescents to the MD [21]. To our knowledge, the dietary patterns of Lebanese adolescents have only been measured using the FFQ (Food Frequency Questionnaire) and the 24 h Recall [11], rather than adopting the scoring method of the KIDMED test. Therefore the aim of our study was to evaluate the association of the AMD with sociodemographic, lifestyle, and anthropometric factors among Lebanese high school adolescents. Specifically, we aimed to analyse in this group the association between low adherence and breakfast intake.

## 2. Materials and Methods

### 2.1. Study Design

A cross-sectional study was carried out on a sample of high school adolescents from private and public schools across Mount Lebanon and Beirut. These two main regions were selected because they included approximately half of the Lebanese students according to the report of the Lebanese Central Administration of Statistics [22].

### 2.2. Sampling Method

Data from the Ministry of Education were used to compile a sampling frame of all schools in the studied regions. The schools were randomly selected based on the stratified cluster sampling method, the strata being public and private schools, whereas the clusters were the secondary classes, and the end units were all the students aged between 15 and 18, and willing to participate. 607 adolescents from grades 10 to 12 were selected, but participants with missing information and medical reasons were excluded from the analysis (7 adolescents).

### 2.3. Ethical Considerations

The study was conducted in accordance with the Declaration of Helsinki (1964). Before the study started, all school directors and adolescents gave written consent. They were informed about the research objectives and had the right to withdraw from the study at any moment. Before anthropometric measurements were carried out, verbal assent was also obtained from each adolescent. Anonymity and confidentiality of participants were respected. Our study does not expose participants to any risk.

### 2.4. Questionnaire

The questionnaire included 20 questions and was self-administered. The sociodemographic data informed us on the age, nationality, place of residence, work status, and the level of education of the students. The medical history included questions about diseases, medications, or supplements. The socioeconomic status (SES) was based on the type of school. Lebanese public school students belonged to low SES due to their lower income and lower school expenses compared to the private schools. The lifestyle characteristics involved the practice of three specific levels of physical activity (PA), based on the frequency and duration: low (<60 min/day), moderate (60–90 min/day), and high (>90 min/day) [23].

The school performance of the students was measured by the question: “What has been your average grade during this year?” with the answers varying between lower than 10 and higher than 15 over 20. We considered grades lower than 10 as a low school performance, grades between 10 and 15 as moderate, and grades higher than 15 as a high school performance.

The assessment of breakfast intake was also evaluated in the questionnaire: by the frequency of intake that was divided into 3 categories: 6–7 days/week; 3–5 days/week; and 0–2 days/week; by the time of intake divided into 2 categories: between 5 am and 10 am, and after 10 am; and finally by the type of foods consumed at breakfast. Subjects that did not consume any food or beverage at breakfast, excluding water, were categorized as breakfast skippers. The types of food choices at breakfast were later on divided into two categories according to the quality of breakfast, as defined by the study of O'Neil et al. [24–26]: a healthy breakfast included three main elements: a cereal food and preferably whole grain products (bread, crackers, ready-to-eat cereals…), a dairy product (milk, yoghurt, cheese…), and a fruit (raw fruit or a fruit juice without added sugar), whereas an unhealthy breakfast included fatty foods and added sugars such as the bakery and confectionary products.

### 2.5. Anthropometric Assessment

Anthropometric measurements were carried out by trained dietitians and taken through calibrated equipment. Participants were weighed to the nearest 0.1 kg, barefoot, using a digital body weight scale (Oregon, BW121, China) and height was measured by using a stadiometer. BMI was calculated by dividing weight in kilograms (kg) by the square of height in meters (m) according to the WHO [27], in order to calculate the BMI-for-age percentiles using the CDC growth charts [28]. According to the CDC classifications, students with measures located between the 5^th^ and the 85^th^ percentile are considered to have a normal weight. However, percentiles lower than 5^th^ are considered as underweight, percentiles between 85^th^ and 95^th^ are considered as overweight, and percentiles ≥95^th^ are considered as obese [28].

### 2.6. Dietary Assessment

Information on dietary intake was gathered by a face-2-face interview with dietitians, through 2 methods: the 24 h-recall and the Food Frequency Questionnaire (FFQ). Particular attention was paid to the factors that maintain the quality of the reported data, such as the nondirectivity of the survey and the estimation of portion size; the latter was defined for each food item and represented one standard serving expressed in household measures including measuring cups, spoons, and graphical representation of food products.

The 24 h recall was used to assess all the foods and beverages consumed in the 24 hours preceding the interview (which constitutes a typical day of the week), stating their amounts and the methods of preparation. As for the FFQ, it referred to the dietary intake of the previous 6 months and included sixty items of foods and beverages commonly consumed in Lebanon. The questionnaire was inspired by models already used in Lebanese studies and abroad [29, 30]. Participants were asked to record the frequency of consumption either per day, per week, per month, or never. The data collected from the FFQ were used to assess the AMD. This partly open-ended approach for recording frequency of consumption allows more flexibility compared with the multiple-choice frequency approach and contributes to the reduction of misclassification errors [30].

To estimate the daily energy and nutrients intake, as well as the nutrients intake at breakfast, we used the professional nutritional software Nutrilog 3.01 [31] based on the Food Composition Tables for Use in the Middle East [32] and the United States Department of Agriculture (USDA) [33]. Those in whom the estimated energy intake was <500 or >5000 kcal were excluded from analysis. Other nutrients were also taken into consideration in order to properly compare a healthy and an unhealthy breakfast, including fibers, added sugars, and cholesterol intake.

### 2.7. Assessment of the Mediterranean Dietary Patterns

The degree of AMD was assessed using the KIDMED index. It is characterized by a total of 16 questions answered by yes or no: “Do you consume one fruit or fruit juice every day?” “Do you consume a second fruit every day?” “Do you consume fresh or cooked vegetables once a day?” “Do you consume fresh or cooked vegetables more than once a day?” “Do you consume fish at least 2–3 times per week?” “Do you go to a fast food restaurant more than once per week?” “Do you consume pulses more than once per week?” “Do you consume rice or pasta 5 times or more per week?” “Do you consume cereal products (bread, cornflakes, etc.) at breakfast?” “Do you consume nuts at least 2–3 times per week?” “Do you usually use olive oil with your meals?” “Do you usually skip breakfast?” “Do you consume dairy products (milk, cheese, etc.) at breakfast?” “Do you consume commercially baked goods or pastries at breakfast?” “Do you consume 40 g of cheese or yoghurt daily?” “Do you have sweets and candy several times every day?” Questions denoting a negative aspect in the report to the Mediterranean diet were scored −1 and those with a positive aspect were scored +1. The KIDMED index ranged from 0 to 12. Subjects with KIDMED scores ≤3 are considered having a poor AMD, those with scores between 4 and 7 have a moderate adherence, and those with scores ≥8 have a high AMD [21].

### 2.8. Statistical Analysis

Sociodemographic, lifestyle characteristics, eating habits, and anthropometric measurements were evaluated by means and percentages for continuous and categorical variables, respectively. To evaluate the associations between variables, the chi-square test was used for categorical variables and ANOVA and Kruskal–Wallis for continuous variables. We further applied linear regression analysis evaluating the association between the levels of AMD (as assessed by the KIDMED scores) and the determinants (age, gender, school type, level of education, school grades, BMI percentiles, and breakfast quality). The analyses were performed using the SPSS 21.0 program (SPSS Inc., Chicago, Illinois). The level of significance was set at *p* < 0.05.

## 3. Results

### 3.1. Population Characteristics

The final sample considered for this study included 600 adolescents (268 boys and 332 girls), aged between 15 and 18 years old. The distribution of this sample was equal across schools with 9 public (50%) and private schools (50%) being studied. Only 17.4% of the students reported working after school, and only 13% had a low academic performance (grades < 10). The majority of the adolescents had low (27.3%) or moderate (31%) PA and were of normal weight (68.1%). 8.3% of the students were obese, and 21.6% were overweight.

### 3.2. Sociodemographic, Lifestyle, Anthropometric Characteristics, and AMD

The associations between KIDMED scores and the participant's characteristics are presented in Table 1. Out of 600 participants, 84.2% of the adolescents had a low (43%) and moderate (41.2%) AMD, with significantly higher prevalence rates being noted among girls compared with boys. In fact, the low AMD group had a higher percentage of boys (53.9%), whereas the moderate and high AMD groups had a higher percentage of girls (61.5% and 64.2%, respectively) compared to boys (38.5% and 35.8%, respectively).

As shown in Table 1, significant differences in index scores were observed between school types. Adolescents from public schools (92.6%) seemed to have a higher AMD than their counterparts (7.4%). Surprisingly, the level of PA in our sample was not significantly associated with the AMD, and neither was the work status. However, the level of education differed significantly between groups, Table 1 shows that 47.4% of the high AMD group belongs to grade 10, while only 18.9% belongs to grade 12. School grades were also significantly associated with the level of adherence, with students having the highest grades (>15), presenting a high level of AMD (25.3%), compared to the low (10.5%) and moderate AMD (15.8%) groups; while the low AMD group presented the highest percentage of students with low grades (17.8%).

Moreover, anthropometric measurements were shown to be significantly associated with the KIDMED scores. Students with low AMD presented the highest mean weight (69.60 ± 0.9 kg) compared to the other two AMD groups. As for the BMI-for-age percentile, 81.1% of students with high index scores presented a normal weight, while the highest percentage of obese and overweight students were observed in the low AMD group (15.5% and 27.5%, respectively), compared to the other two groups.

### 3.3. Breakfast Intake, Breakfast Quality, and AMD

In addition to the overall breakfast intake, specific breakfast options were included in our study in order to evaluate the association between this meal's quality and the AMD. Out of 600 participants, 52.8% were breakfast consumers and 47.16% were breakfast skippers. While among breakfast consumers, 68.4% had a healthy breakfast and 31.5% had an unhealthy breakfast.

In the first step, we compared the level of AMD among breakfast consumers and nonconsumers. We noticed that adolescents having a moderate and high AMD were more likely to be breakfast consumers (68.8% and 71.6%, respectively) than skippers (31.2% and 28.4%, respectively). Among breakfast skippers, 69.4% had a poor adherence while only 30.6% of breakfast consumers were poorly adherent to the Mediterranean diet (*P* < 0.001).

In the second step, breakfast consumers were divided into two categories based on the quality of the meal consumed [24] (Table 2). When comparing breakfast quality between the groups of AMD in Table 2, we noted that subjects who consumed a healthy breakfast had higher index scores (healthy breakfast (65.3%) vs. breakfast skippers (28.4%) vs. unhealthy breakfast (6.3%)), while breakfast skippers had the lowest scores compared to the other groups (breakfast skippers (69.4%) vs. unhealthy breakfast (17.4%) vs. healthy breakfast (13.2%)) (*P* < 0.001).

### 3.4. Energy, Nutrients Intake, and AMD

Table 3 examines the daily energy and nutrients intake means, reflecting their association with the overall AMD, and for boys and girls separately. Energy intake was significantly higher in boys who have a high AMD compared to those with poor AMD (1658.4 ± 82.5 kcal/day vs. 1531.5 ± 49.8 kcal/day) (*P* < 0.05), while no significant difference was observed in girls. Total carbohydrates intake showed a significant result for boys and for the total sample, respectively, where the subjects having a moderate (210.5 ± 7.7 g/day; 195.4 ± 4.5 g/day) and high AMD (214.3 ± 13.1 g/day; 198.1 ± 6.6 g/day) presented a significantly high intake of carbohydrates. The protein intake was significantly higher in subjects with high AMD for boys and girls, compared with the ones having a poor AMD. As for the total lipids intake, no significant difference was observed in all groups; nor did the 2 types of fatty acids (MUFA and PUFA) change significantly between the studied groups. The lipids intake (% DEI) was significantly lower in subjects with high AMD for boys and for the total sample. Also, the SAFA intake was significantly lower in subjects with high AMD for girls and for the total sample. In boys, the fiber intake was significantly higher for ones having a moderate and high AMD compared to those having a low AMD; it was not significant for girls. Finally, the water intake was significantly higher in subjects having a high AMD for both genders and for the total sample.

### 3.5. Predictor Variables of AMD

The results of the multiple regression model predicting the KIDMED scores are shown in Table 4. School grades, school type, BMI percentiles, and the type of breakfast were the only significant predictors of the KIDMED scores (*P* < 0.05) in a model that accounts for 30.2% of the overall variance. Regarding the participant characteristics, school grades were positively associated with the AMD (standardized *β*=0.096; *P*=0.048), while a negative association was observed for the BMI percentiles (standardized *β*=−0.101; *P*=0.044). Furthermore, the school type was also correlated to the index scores; students attending public schools had a significant higher AMD than those attending private schools. As regards for breakfast quality, a healthy breakfast was related to a greater AMD than an unhealthy breakfast.

## 4. Discussion

Since the adolescence is a critical period during which students may adopt lasting health behaviors, the present transition in the dietary pattern adopted by this group of age is a critical aspect to monitor. In our study, we evaluated the AMD and its association with lifestyle characteristics, anthropometric measurements, breakfast, and nutrients intake. The majority of our adolescents had a low and moderate AMD, with significantly higher adherence observed among girls compared with boys. Our study is one of many others highlighting the gender differences in the adoption of the MD [11, 34], while no statistical difference was found in others [13, 16]. When comparing the scores of our sample with other studies to assess the AMD in the Mediterranean region (Table 5), our population presented the highest proportion of adolescents having a low AMD, followed by the adolescents of Greece (42%), Turkey (40.6%), and Italy (32.8%) [13, 16, 17]. On the contrary, Spain and Southern Spain seemed to have the lowest percentages in this department (12.2% and 2%, respectively), while the highest percentages of adolescents had a high AMD (30.9% and 46.9%, respectively) [18, 19]. Italy, Cyprus, and Greece had the lowest percentages regarding the high AMD (5%, 6.7%, and 6.8%) with more adolescents in this country having a moderate AMD [10, 16, 17].

Among subject's sociodemographic characteristics, age and gender were not significant predictors of the AMD, although there was found to be a significant difference for both variables. These findings are in accordance with other studies that found a negative association between age and KIDMED scores and showed that girls have a higher adherence than boys [19].

The school type however was a strong predictor of the AMD. In fact, students from public schools were found to have a high AMD compared to their counterparts, which seems to be due to the SES of the students, knowing that students in public schools tend to come from low-income families due to the differences between public and private schools expenditures [35]. In fact, there are conflicting results in the studies evaluating the association between the SES and the dietary habits, which may be due to the disparities between developing and industrialized countries [36]. Studies conducted in Lebanon on older adults showed that subjects with higher income made healthier choices in their dietary pattern, thus having the purchasing power needed to access healthier food [37]. However, when evaluating the SES with childhood and adolescent's obesity and dietary habits, Nasreddine et al. showed that in middle-income countries such as Lebanon, pediatric obesity appears to be predominant among high-income families [38].

Regarding the anthropometric measurements, our study found that adiposity is associated with the AMD, with the majority of our overweight and obese students presenting a low AMD. The linear regression model conducted showed a negative association between BMI percentiles and KIDMED scores proving that a higher body weight is correlated with a lower AMD. This result constitutes further evidence for this hypothesis found in several studies, conducted in different Mediterranean countries with low AMD, suffering from high rates of pediatric obesity, such as Italy, Cyprus, and Greece [10, 16, 39]. In fact, Fahed et al. reported that the transition from traditional MD to western dietary patterns increased the prevalence of metabolic diseases and obesity in Mediterranean countries [40].

Likewise, school performance was also proved by this study to be positively associated with the AMD. In fact, school grades were positively correlated with the KIDMED scores, where students having higher school grades presenting higher scores. Studies evaluating the association between the MD and cognition are usually carried out on older adults and elderly [41, 42]. However, few studies have evaluated the impact of the MD on adolescents' academic performance, for example, the study of Esteban-Cornejo et al. conducted on Spanish adolescents and the study of Santomauro et al. conducted on Italian adolescents [43, 44]. The results of both studies agree with our findings on the beneficial impact of the MD on adolescents' school performance.

Among the 16 questions of the KIDMED test, 4 of them refer to the breakfast intake and quality, which is the reason why we evaluated the association between breakfast and MD. The results of the study sample showed that all students belonging to the high adherence group were breakfast consumers, while breakfast skippers had a low AMD, which was also concluded in several studies, for example, the study of Lazarou et al. conducted on the adolescents of Cyprus [10]. Less is, however, known about the relationship between the breakfast quality and the AMD. In our study, the breakfast quality was a strong predictor of the AMD. We found that healthy breakfast consumers had a higher AMD. Based on our knowledge, no other study has sought this association, but studies have shown that breakfast skippers tend to have poor dietary habits overall, with an irregular snacking frequency and a high consumption of unhealthy foods and beverages [45, 46].

Regarding daily nutrients intake, our study provided evidence that students having a high AMD had a higher protein, fiber, and water intake compared to the other AMD groups. These findings are in agreement with several studies [37, 46] and may be explained by the high intake of healthy animal and vegetable sources of protein in the MD, such as fish, dairy products, legumes, and nuts; and the high intake of legumes and whole grain cereals as a source of fiber [3]. Carbohydrates intake were higher among students with high AMD, which is mostly due to the different varieties of carbohydrate sources present in this pattern, knowing that the basic elements composing the three main meals in the MD are fruits, vegetables, and whole grain cereals [3]. Finally, fat intake was higher among the low-adherence group, with significant high intake of SAFA. These findings are similar to those of a study conducted on a national sample of Lebanese adolescents, showing that subjects adopting a western dietary rather than the traditional Mediterranean dietary pattern were associated with a higher intake of high fat foods and a higher frequency of eating out [11].

Overall, our study has shown that the prevalence of Lebanese adolescents following an MD is decreasing and a transition to a Western dietary pattern is predominating in our youth. The reason behind the decreased adherence in Lebanese adolescents is mainly due to the increased availability of non-Mediterranean food products during the past years [12] and the adoption of a western-like dietary pattern by the adolescents of our country [11, 12].

There are few limitations in this study: first, since this study is retrospective and cross-sectional, we could not determine the causality of this association, only the interrelationships. However, based on our knowledge, it is the first study evaluating the dietary pattern of Lebanese adolescents according to the standards of the traditional MD, using the KIDMED index. Therefore, it should call for future studies and could be used for further investigations. Second, the questionnaires were self-administered which may lead to misreporting and data bias; but dietitians were fully aware of the situation and monitored the students intensively. Third, the daily nutrients intake data are based on a 24 h recall for only one day, which may not be representative of the whole week, but as mentioned before, it represented a typical day of the week. Finally, the study is conducted in only two regions of Lebanon, which may not give us the ability to consider our results as a conclusion on a national basis, but it can give us an idea of the present rating regarding the AMD and obesity and offers data for further studies, knowing that they included approximately half of the Lebanese students.

## 5. Conclusions

In conclusion, this study showed a low adherence (43%) to the MD among Lebanese adolescents. Students with higher AMD had better school performance and lower adiposity. In addition, the school type was found to be a significant predictor of the KIDMED scores, with public schools having the highest adherence. Our results have also provided evidence on the association between the AMD and a healthier dietary intake and breakfast quality. Finally, given that the dietary patterns adopted in the early years of life can lead to an increasing risk of metabolic diseases in adulthood, our data may raise further need for increased awareness on the benefits of the MD among parents and adolescents in Lebanon through school-based interventions.

## Figures and Tables

**Table 1:** Association between sociodemographic, lifestyle, anthropometric characteristics, and adherence to the Mediterranean diet among Lebanese adolescents, *N*=600.

Variables	AMD			*p* value^1^
	Low (0–3) (*n* = 258) *n* (%)	Moderate (4–7) (*n* = 247) *n* (%)	High (≥8) (*n* = 95) *n* (%)	
Sociodemographic and lifestyle characteristics				
Gender				
Boys	139 (53.9)	95 (38.5)	34 (35.8)	<0.001^*∗*^
Girls	119 (46.1)	152 (61.5)	61 (64.2)	
School type				
Private	160 (62.0)	133 (53.8)	7 (7.4)	<0.001^*∗*^
Public	98 (38.0)	114 (46.2)	88 (92.6)	
Work status				
Yes	47 (18.2)	34 (13.8)	23 (24.2)	0.065
No	211 (81.8)	213 (86.2)	72 (75.8)	
Level of education				
Grade 10	81 (31.4)	89 (39.0)	45 (47.4)	0.015^*∗*^
Grade 11	85 (32.9)	76 (32.8)	32 (33.7)	
Grade 12	92 (35.7)	82 (28.2)	18 (18.9)	
School grades				
<10	46 (17.8)	21 (8.5)	11 (11.6)	<0.001^*∗*^
11–15	185 (71.7)	187 (75.7)	60 (63.1)	
>15	27 (10.5)	39 (15.8)	24 (25.3)	
Level of physical activity^2^				
Low	74 (44.6)	63 (36.8)	27 (41.5)	0.631
Moderate	70 (42.2)	85 (49.7)	31 (47.7)	
High	22 (13.3)	23 (13.5)	7 (10.8)	

Anthropometric characteristics (mean ± SE)				
Weight (kg)	69.60 ± 0.900	61.73 ± 0.720	63.38 ± 1.170	<0.001^*∗*^
Height (m)	1.70 ± 0.006	1.68 ± 0.006	1.67 ± 0.007	0.008^*∗*^
BMI, *N* (%)				
Underweight	5 (1.9)	6 (2.4)	0 (0.0)	<0.001^*∗*^
Normal weight	142 (55.0)	190 (76.9)	77 (81.1)	
Overweight	71 (27.5)	45 (18.2)	14 (14.7)	
Obese	40 (15.5)	6 (2.4)	4 (4.2)	

BMI: body mass index. ^1^The difference between groups was examined using the chi-square test for categorical variables and ANOVA test for continuous variables. ^2^A low level of physical activity (LPA) corresponds to a PA < 60 min/week; a moderate LPA corresponds to a PA between 60 and 90 min/week; a high LPA corresponds to a PA > 90 min/week. ^*∗*^*p* < 0.05 is considered as significant.

**Table 2:** Association between type of breakfast consumers, nonconsumers, and adherence to the Mediterranean diet among Lebanese adolescents, *N*=600.

	AMD			*p* value^1^
	Low (0–3) *n* (%)	Moderate (4–7) *n* (%)	High (≥8) *n* (%)	
Healthy breakfast consumers^a^	34 (13.2)	121 (49.0)	62 (65.3)	<0.001^*∗*^
Unhealthy breakfast consumers^b^	45 (17.4)	49 (19.8)	6 (6.3)	
Breakfast skippers^c^	179 (69.4)	77 (31.2)	27 (28.4)	

^1^Difference between breakfast groups was obtained using the chi-square test for categorical variables. ^*∗*^*p* < 0.05 was considered significant. ^a^Composed from a cereal product (bread, rusk, and slightly sweet cereals), a dairy product (labneh, milk, yoghurt, cheese, including their fat content is ≤ 5 g), or a fruit compost or half a glass of unsweetened fruit juice or a half cup of raw vegetables. ^b^Do not contain the elements of healthy breakfast. ^c^Breakfast skippers: breakfast consumption for 0–2 days/week only; never or after 10 am.

**Table 3:** Association between daily energy, nutrients intake means, and adherence to the Mediterranean diet by gender, among Lebanese adolescents, *N*=600.

	Boys *n*=268			*P* ^1^	Girls *n*=332			*P* ^1^	Total sample *n*=600			*P* ^1^
	AMD				AMD				AMD			
	Poor	Moderate	High		Poor	Moderate	High		Poor	Moderate	High	
	Mean ± SE	Mean ± SE	Mean ± SE		Mean ± SE	Mean ± SE	Mean ± SE		Mean ± SE	Mean ± SE	Mean ± SE	
DEI	1531.5 ± 49.8	1855.2 ± 147.2	1658.4 ± 82.5	0.044^*∗*^	1489.4 ± 51.7	1521.3 ± 37.2	1495.4 ± 44.7	0.591	1512.1 ± 36.1	1649.7 ± 61.6	1553.8 ± 41.7	0.113
Total CHO (g/day)	176.0 ± 6.5	210.5 ± 7.7	214.3 ± 13.1	0.001^*∗*^	182.1 ± 7.1	186.0 ± 7.1	189.0 ± 7.1	0.804	179.0 ± 4.8	195.4 ± 4.5	198.1 ± 6.6	0.017^*∗*^
CHO (% DEI)	45.6 ± 1.0	56.2 ± 1.2	64.5 ± 1.5	0.065	48.9 ± 1.0	51.7 ± 0.9	50.3 ± 1.2	0.737	47.8 ± 0.7	49.3 ± 0.7	50.8 ± 0.9	0.062
Total pro (g/day)	60.7 ± 2.4	71.7 ± 2.9	67.6 ± 4.6	0.014^*∗*^	59.3 ± 2.2	58.4 ± 1.7	65.9 ± 2.5	0.073	60.1 ± 1.6	63.6 ± 1.6	66.5 ± 2.3	0.076
Proteins (% DEI)	16.6 ± 0.5	17.0 ± 0.6	16.8 ± 1.2	0.855	16.6 ± 0.5	15.8 ± 0.4	18.1 ± 0.7	0.019^*∗*^	16.6 ± 0.4	16.3 ± 0.4	17.6 ± 0.6	0.163
Total lip (g/day)	65.0 ± 2.9	65.9 ± 2.8	59.9 ± 4.1	0.529	59.6 ± 2.8	60.7 ± 2.2	54.4 ± 2.6	0.305	62.5 ± 2.1	62.8 ± 1.7	56.4 ± 2.2	0.156
Lipids (% DEI)	37.4 ± 0.9	34.5 ± 0.9	32.3 ± 1.2	0.008^*∗*^	35.0 ± 0.9	35.4 ± 0.8	32.3 ± 1.1	0.095	36.3 ± 0.6	35.1 ± 0.6	32.3 ± 0.8	0.003^*∗*^
SAFA (% DEI)	15.2 ± 0.5	13.8 ± 0.6	13.3 ± 0.8	0.130	14.4 ± 0.5	14.3 ± 0.5	12.1 ± 0.5	0.015^*∗*^	14.8 ± 0.4	14.1 ± 0.4	12.5 ± 0.4	0.004^*∗*^
MUFA (% DEI)	12.6 ± 0.4	12.3 ± 0.6	10.8 ± 0.6	0.224	11.7 ± 0.5	12.1 ± 0.4	11.5 ± 0.5	0.680	12.1 ± 0.3	12.1 ± 0.3	11.3 ± 0.4	0.295
PUFA (% DEI)	7.8 ± 1.1	5.7 ± 0.4	5.1 ± 0.4	0.180	5.9 ± 0.3	5.9 ± 0.3	5.7 ± 0.3	0.638	6.9 ± 0.6	5.8 ± 0.2	5.5 ± 0.2	0.117
Total chol (mg/d)	172.8 ± 12.4	223.0 ± 18.8	183.8 ± 24.9	0.195	162.2 ± 12.8	186.1 ± 15.1	195.1 ± 80.8	0.356	166.3 ± 8.8	198.9 ± 11.8	191.1 ± 16.0	0.072
Total fib (g/day)	13.0 ± 0.7	16.4 ± 0.9	16.1 ± 1.2	0.011^*∗*^	15.1 ± 0.8	15.1 ± 0.8	15.3 ± 0.8	0.988	14.0 ± 0.6	15.6 ± 0.6	15.6 ± 0.7	0.089
Water (g/day)	2713.5 ± 56.4	3037.5 ± 77.2	3218.5 ± 125.1	<0.001^*∗*^	2925.0 ± 71.2	2939.5 ± 63.6	3542.1 ± 86.9	<0.001^*∗*^	2811.6 ± 45.2	2977.2 ± 49.1	3426.3 ± 72.9	<0.001^*∗*^

Mod: moderate; DEI: daily energy intake; CHO: carbohydrates; Pro: proteins; Lip: lipids; SAFA: saturated fatty acids; MUFA: monounsaturated fatty acids; PUFA: polyunsaturated fatty acids; Chol: cholesterol; Fib: fibers. ^*∗*^*P* < 0.05 is considered significant. ^1^Difference between groups was obtained using ANOVA and Kruskal–Wallis for continuous variables.

**Table 4:** Multiple regression model predicting KIDMED scores among Lebanese adolescents, *N*=600.

Variables	Total sample	
	Standardized *β*	*P*
Age	−0.072	0.411
Gender	0.058	0.248
School type	0.447	<0.001^*∗*^
Level of education	0.066	0.437
School grades	0.096	0.048^*∗*^
BMI percentiles	−0.101	0.044^*∗*^
Breakfast quality	−0.217	<0.001^*∗*^

^*∗*^ *P* < 0.05 is considered significant.

**Table 5:** Comparison between studies evaluating adolescents' AMD in different Mediterranean countries.

	Country	AMD			
		Age (years)	Low (%)	Moderate (%)	High (%)
Present study	Lebanon	15 to 18	43.0	41.2	15.8
Roccaldo et al. [16]	Italy	13 to 16	32.8	62.2	5.0
Cömert et al. [13]	Turkey	10 to 16	40.6	51.6	7.7
Grao-Cruces et al. [18]	Spain	11 to 18	12.2	56.9	30.9
Mariscal-Arcas et al. [19]	Southern Spain	8 to 16	2.0	51.1	46.9
Lazarou et al. [10]	Cyprus	6 to 12	29.0	55.5	6.7
Chatzi et al. [15]	Crete	6 to 18	27.9	43.8	28.3
Mazaraki et al. [17]	Greece	12 to 17	42.0	51.2	6.8

## Data Availability

The data used to support the findings of this study are available from the corresponding author upon reasonable request.
